# Supplementary material for: Genetic Structure and Genetic Diversity of the Endemic Korean Aucha Perch, Coreoperca herzi (Centropomidae), in Korea
Source: Animals (Basel). 2023 Aug 14;13(16):2614. doi: 10.3390/ani13162614 (PMC10451468; doi:10.3390/ani13162614)
Supplement: Supplementary file 1 [file animals-13-02614-s001.zip › animals-2497200-supplementary.pdf]

Table S1. Sampling sites and number of individuals in the study

Location	Code	Water system	N	Location
Namgang River	NNG	Nakdonggang River	12	35°31'50.43″ N, 127°47'00.50" E
Deokcheongang River	NDC	Nakdonggang River	13	35°15'23.74″ N, 127°53'46.29" E
Seokgyocheon Stream	NSG	Nakdonggang River	2	35°14'41.04″ N, 128°21'31.48" E
Danjangcheon Stream	NDJ	Nakdonggang River	16	35°30'57.44″ N, 128°52'53.14" E
Imcheon Stream	NIM	Nakdonggang River	5	35°28'48.80″ N, 127°48'29.30" E
Yangsancheon Stream	NYS	Nakdonggang River	6	35°19'30.00″ N, 129°01'14.00" E
Dalcheon Stream	HDC	Hangang River	41	36°36'54.73″ N, 127°42'06.20" E
Sangcheoncheon Stream	HSC	Hangang River	22	37°45'02.71″ N, 127°26'06.75" E
Hongcheongang River	HHC	Hangang River	10	37°42'27.00″ N, 127°50'20.00" E
Wonseocheon Stream	HWS	Hangang River	7	37°12'39.88″ N, 127°57'51.20" E
Miwoncheon Stream	HMW	Hangang River	9	37°40'53.00″ N, 127°29'09.00" E
Ssangcheon Stream	HSSC	Hangang River	16	36°47'23.89″ N, 127°51'13.86" E
Jeonjucheon Stream	MJJ	Mangyeonggang River	11	35°48'31.71″ N, 127°09'48.03" E
Yudeungcheon Stream	GYD	Geumgang River	8	36°18'13.48″ N, 127°22'52.75" E

Namdaecheon Stream	GND	Geumgang River	7	36°00'25.00″ N, 127°40'41.00" E
Onamcheon Stream	SON	Seomjingang River	8	35°00'28.00″ N, 127°06'26.00" E
Seomjingang River	SSJ	Seomjingang River	7	35°42'42.25″ N, 127°18'31.85" E
Jiseokcheon Stream	YJS	Yeongsangang River	11	34°57'28.91″ N, 126°58'31.86" E
Yangyang Namdaecheon Stream	YYND	Yangyang Namdaecheon Stream	8	37°57'11.00″ N, 128°39'44.00" E

N: Number of samples.
